# Psychometric evaluation of the muscle dysmorphic disorder inventory (MDDI) among gender-expansive people

**DOI:** 10.1186/s40337-022-00618-6

**Published:** 2022-07-06

**Authors:** Emilio J. Compte, Chloe J. Cattle, Jason M. Lavender, Tiffany A. Brown, Stuart B. Murray, Matthew R. Capriotti, Annesa Flentje, Micah E. Lubensky, Juno Obedin-Maliver, Mitchell R. Lunn, Jason M. Nagata

**Affiliations:** 1 Eating Behavior Research Center, School of Psychology, Universidad Adolfo Ibáñez, Santiago, Chile; 2 Research Department, Comenzar de Nuevo Treatment Center, Monterrey, Mexico; 3 Department of Pediatrics, University of California, San Francisco, 550 16th Street, 4th Floor, Box 0110, San Francisco, CA 94158 USA; 4 Military Cardiovascular Outcomes Research Program (MiCOR), Department of Medicine, Uniformed Services University of the Health Sciences, Bethesda, MD USA; 5 The Metis Foundation, San Antonio, TX USA; 6 Department of Psychological Sciences, Auburn University, Auburn, AL USA; 7 Department of Psychiatry and Behavioral Sciences, University of Southern California, Los Angeles, CA USA; 8 Department of Psychology, San José State University, San Jose, CA USA; 9 The PRIDE Study/PRIDEnet, Stanford University School of Medicine, Stanford, CA USA; 10 Department of Community Health Systems, University of California, San Francisco, San Francisco, CA USA; 11 Alliance Health Project, Department of Psychiatry and Behavioral Sciences, University of California, San Francisco, San Francisco, CA USA; 12 Department of Obstetrics and Gynecology, Stanford University School of Medicine, Stanford, CA USA; 13 Department of Epidemiology and Population Health, Stanford University School of Medicine, Stanford, CA USA; 14 Division of Nephrology, Department of Medicine, Stanford University School of Medicine, Stanford, CA USA

**Keywords:** Gender-expansive, Non-binary, Transgender persons, Genderqueer, Muscle dysmorphia, MDDI

## Abstract

**Purpose:**

Muscle dysmorphia is generally classified as a specific form of body dysmorphic disorder characterized by a pathological drive for muscularity and the preoccupation that one is too small or not sufficiently muscular. The majority of research on the condition has been conducted in cisgender men with a paucity of literature on gender minority people, a population that is at risk for muscle dysmorphia. One of the most widely used measures of muscle dysmorphia symptoms, the Muscle Dysmorphic Disorder Inventory (MDDI), has not been psychometrically validated for use in gender minority samples, the aim of the present study.

**Methods:**

We evaluated the psychometric properties of the MDDI in a sample of 1031 gender-expansive individuals (gender minority people whose gender identity differs from that assumed for their sex assigned at birth and is not exclusively binary man or woman) aged 18–74 who were part of The PRIDE Study, a large-scale, U.S., longitudinal cohort study.

**Results:**

Using a two-step, split-sample exploratory and confirmatory factor analytic approach, we found support for the original three-factor structure of the measure. The subscales showed adequate internal consistency, and convergent validity was supported based on significant associations of the MDDI subscale scores with theoretically related scores on a widely used measure of disordered eating.

**Conclusions:**

These findings provided novel support for adequate psychometric properties of the MDDI in a sample of gender-expansive individuals, facilitating the use of this measure in future research on muscle dysmorphia in this understudied and at-risk population.

## Plain English Summary

Gender-expansive describes gender identities that do not fit within the binary gender identity system, such as man or woman. We asked gender-expansive participants in The PRIDE Study to fill out a widely used survey about muscle dysmorphia (when someone is worried about not being muscular enough). We found that this issue about muscularity has three parts for gender-expansive people: (1) a desire to be bigger and more muscular, (2) dissatisfaction with the way their body looks, and (3) problems with normal life functions. We find that this survey is appropriate for use in gender-expansive people. These results can help providers and researchers understand the muscle-related problems that gender-expansive people face.

## Introduction

Muscle dysmorphia is generally classified as a specific form of body dysmorphic disorder characterized by an extreme drive for muscularity and preoccupation with the idea that one is too small or not sufficiently muscular [1, 2]. Muscle dysmorphia is associated with significant distress and psychosocial impairment as well as a myriad of pathological behaviors including excessive exercise, disordered eating, and the use of appearance- and performance-enhancing substances such as anabolic androgenic steroids [1–7]. Comorbidity with other forms of psychopathology is also common, and those with muscle dysmorphia have been found to have an increased risk for eating disorders, anxiety, and depression [3, 8, 9] as well as elevated rates of substance use and suicide [4]. Prevalence estimates vary dramatically across specific populations, including 1.4% of Australian adolescent girls [10], 6–7% of college men, and nearly 50% of bodybuilding men [7].

Although most research on muscle dysmorphia has been conducted using samples of cisgender men (i.e., individuals who identify as a man and were assigned male at birth), accumulating evidence suggests that gender minority people (i.e., those whose gender(s) differs from that assumed for their sex assigned at birth) are also at risk. Gender minority people face substantial mental health disparities, including elevated rates of overall psychological distress, mood and anxiety disorders, suicidality, and self-injurious behavior [11–13]. The term gender-expansive has been used to describe a spectrum of gender identities that fall outside of the binary structure (i.e., man and woman); this includes various non-binary and genderqueer identities and those who describe multiple or no gender identities. Research suggests that, compared to their exclusively cisgender and binary transgender peers, gender-expansive individuals have the lowest degree of social support and the highest risk of bullying [11]. Moreover, in the United States (U.S.) and United Kingdom, gender-expansive people reported a lower quality of life and increased psychological distress compared to binary transgender and cisgender people [14]. These findings emphasize the importance of characterizing the nature and severity of psychopathology experienced by gender minorities generally, as well as identifying potentially heightened disparities among certain subgroups such as gender-expansive people.

Importantly, with regards to muscle dysmorphia specifically, gender minority people experience greater dissatisfaction with their bodies, increased rates of diagnosed eating disorders, and more restrictive eating behaviors and excessive exercise [15–17]. Indeed, the differences in muscle dysmorphia symptomatology among various gender groups (e.g., cisgender, transgender, and gender-expansive) emphasize the importance of studying the drive for muscularity and the manifestations of muscle dysmorphia in these subgroups [18, 19]. Nagata et al. (2021) found that transgender men reported higher scores on a muscle dysmorphia measure compared to transgender women and non-binary individuals. Similarly, Amodeo et al. (2020) compared cisgender, transgender, and gender non-binary individuals and found that transgender men score higher on appearance anxiety/avoidance.

The Muscle Dysmorphic Disorder Inventory (MDDI) [20] is one of the most used instruments to evaluate symptoms of muscle dysmorphia in clinical and research settings [21]. The measure is comprised of 13 items with three subscales assessing Drive for Size, Appearance Intolerance, and Functional Impairment. In addition to its relative brevity and focus on core symptoms, a particular benefit of the MDDI is its inclusion of the subscale assessing functional impairment, which is a key diagnostic criterion for muscle dysmorphia. Support for the original three-factor structure has been highly consistent across multiple studies [22–29] with only one exception [30]. Santarnecchi and Dèttore found a four-factor structure in a small sample of 60 non-competing presumably cisgender male bodybuilders. Moreover, support has been found for the psychometric properties of the MDDI in samples from numerous countries, including Argentina, Turkey, Spain, Brazil, Norway, and Germany [22–28]. To date, however, most studies have been conducted in samples of cisgender, or presumed cisgender, men; furthermore, most of these are reported to be weightlifters, bodybuilders, or highly physically active. A recent study did report normative data for the MDDI among a community sample of gender-expansive individuals; however, there is no thorough psychometric evaluation of the MDDI to examine its factor structure and psychometric properties in gender-expansive individuals [19].

To address these gaps in the existing literature, this study aimed to psychometrically validate the MDDI in a large sample of adults from the U.S. with a gender group that is not exclusively cisgender or binary transgender—a population we refer to as gender-expansive. Specifically, exploratory and confirmatory factor analyses were conducted to evaluate the factor structure in this sample. Furthermore, the internal consistency of the MDDI subscales was evaluated, and convergent validity based on associations with theoretically relevant measures of disordered eating was examined. Consistent with numerous replications across other samples, we hypothesized that the original three-factor structure of the MDDI proposed by Hildebrandt et al. (2004) would be supported in the current sample and that the subscales would show adequate internal consistency. In support of convergent validity, and consistent with conceptual associations between the constructs of muscle dysmorphia and disordered eating symptoms, we expected to find significant (positive or negative) associations between scores on the MDDI subscales and scores on relevant subscales from the Eating Disorder Examination-Questionnaire (EDE-Q). Specifically, the MDDI Appearance Intolerance subscale would be positively associated with the EDE-Q Shape Concern and Weight Concern subscales, given their overlapping nature (i.e., body image concerns). The MDDI Functional Impairment subscale would be significantly, positively correlated with the EDE-Q Global Score, given the impairment-related content (e.g., social avoidance, difficulties with concentration) reflected in items from several subscales comprising the Global Score. In contrast, The MDDI Drive for Size subscale would be negatively associated with the EDE-Q Restraint and Weight Concern subscales, given the differential focus on specific behaviors and concerns (i.e., those related to desires to be larger versus those focused predominantly on desires for a lower weight).

## Methods

### Study population, data collection, and recruitment

#### Procedure

The (Population Research in Identity and Disparities for Equality) PRIDE Study [31] is a national (U.S.), longitudinal cohort study of sexual and gender minority adults. Inclusion criteria were: age ≥ 18, identification as a sexual and/or gender minority, living in the U.S. or its territories, and the ability to respond to questionnaires written in English. Recruitment efforts, led by PRIDEnet (a national network of individuals and organizations formed to engage sexual and gender minorities), included online advertising via social media and newsletters, word-of-mouth, event outreach, and distribution of branded promotional materials. The study was approved by the Institutional Review Boards of the University of California, San Francisco and Stanford University. All participants provided written informed consent and compensation was not provided. Further details of the study, including design, population demographics, and description of the digital platform, have been described elsewhere [31]. Participants in The PRIDE Study were invited to complete an online questionnaire, the Eating and Body Image Survey, from April to August 2018.

#### Participants

Participants were asked about their gender identity (“What is your current gender identity?”) and were able to choose more than one option and write in their identity if it was not provided in the preset categorical answer choices. They were asked to identify the sex assigned to them at birth (“What sex were you assigned at birth on your original birth certificate?”). For this study, we excluded those participants who were classified exclusively as a cisgender man (gender identity: man, assigned sex at birth: male), cisgender woman (gender identity: woman, assigned sex at birth: female), transgender man (gender identity: man, assigned sex at birth: female), and/or transgender woman (gender identity: woman, assigned sex: male). Participants who selected “genderqueer,” multiple gender identities, “another gender identity,” and/or provided a write-in (e.g., non-binary, nonconforming, genderfluid, agender, and bigender) were considered gender-expansive and included in the present study. Of the 4672 participants from The PRIDE Study who completed the Eating and Body Image Survey, 1120 were classified as gender-expansive people. In addition to data on gender identity and sex assigned at birth, participants self-reported sociodemographic data including age, race/ethnicity, educational status, weight, and height (the latter two of which were used to calculate body mass index [BMI; kg/m^2^]).

Of the total sample, 89 participants had more than 50% of their values missing and were excluded from the analyses. The final sample was comprised of 1031 gender-expansive participants with a mean age of 29.9 years (*SD* = 9.8, range = 18–74.3) and a mean BMI of 28.7 kg/m^2^ (*SD* = 8.53, range = 12.9–70.8). Furthermore, 72.3% of the participants identified as White, 2.6% as Asian, 1.0% as Black, 0.3% as Native American/American Indian, 11.0% as another race, 2.4% as multi-race (e.g., reported two or more racial identities), and 10.4% did not report their race. Additionally, 5.4% of participants identified as Hispanic, Latino, or Spanish in origin. From the total sample, 82.8% were assigned female sex at birth, 13.1% were assigned male sex at birth, and 4.1% did not report sex assigned at birth. Finally, 58.1% of participants reported having a college degree or higher, and 87.6% were born in the U.S.

#### Measures

*Muscle Dysmorphic Disorder Inventory* (MDDI) [20]. The MDDI is a 13-item measure that assesses symptoms of muscle dysmorphia. Items are rated on a five-point Likert-type scale (1 = *never*; 5 = *always*), and higher scores indicate greater symptom severity. The MDDI is comprised of three subscales: *Drive for Size* (DFS), *Appearance Intolerance* (AI), and *Functional Impairment* (FI). In this study, item 5 *(“I think my chest is too small”*) was modified to specify “chest (muscle)”, so as to not confuse “chest” with breast size [29]. Previous studies across diverse populations have supported the original three-factor structure, including a recent publication that found good psychometric properties among cisgender gay men and lesbian women [29]. Norms for the MDDI have been published in a variety of populations [32] including among gender-expansive individuals [19]. Internal consistency values for the MDDI subscales in the current sample are presented in Table 2.

*Eating Disorders Examination-Questionnaire* (EDE-Q) [33]. The EDE-Q is a self-report measure of eating disorder attitudes and behaviors experienced over the previous 28 days. The EDE-Q provides four subscale scores*: Restraint* (five items), *Eating Concern* (five items), *Weight Concern* (five items), and *Shape Concern* (eight items). The Global Score is calculated as the average of the four subscales. Attitudinal items are rated on a seven-point ordered scale with higher scores reflecting greater eating disorder symptom severity. Norms for the EDE-Q have been published in a variety of populations [34, 35] including among gender-expansive individuals [36]. Internal consistency values for the EDE-Q subscales and Global Score are presented in Table 2.

### Data analysis

Among those included in the analysis, 0.07% of missing values were observed, and the nonparametric test of homoscedasticity suggested that the mechanism was consistent with data missing completely at random (*p* = 0.29); consequently, data imputation was performed using multivariate imputation by chained equations. Descriptive statistics were reported as means and standard deviations (SD) for continuous variables and as percentages for categorical variables. Following recent guidelines in scale validation [37, 38], the full sample of gender-expansive participants was randomly divided in a 1:1 ratio into split-half subsamples. An exploratory factor analysis (EFA) was conducted to determine the underlying factor structure of the MDDI using data from the first split-half subsample, after which confirmatory factor analysis (CFA) was conducted to assess the retained EFA model using data from the second split-half subsample. Because accurate sample adequacy for EFA is best determined after data analyses (communalities ≥ 0.50), guidelines to recruit as large a sample as possible were followed [38]; however, a minimum sample size of 260 participants was considered suitable, offering a 20:1 ratio per item [39]. To determine sample size requirements for the CFAs, a power analysis based on an RMSEA value consistent with a good model fit [40] was conducted for the original 13-item, three-factor model. A minimum sample size of 209 participants was required for a power of 0.80, an RMSEA value of 0.05, and an alpha level of 0.05.

Given that the assumption of multivariate normality was not fulfilled for the first split-half subsample (Mardia's Skewness 4419.11, *p* < 0.001), EFA was based on the principal-axis factoring estimation method [41]. Factors were assumed to be correlated, and the non-orthogonal Oblimin rotation was used. The Kaiser–Meyer–Olkin (KMO) measure of sampling adequacy and Bartlett’s test of sphericity were used to determine if the data met the assumptions for an EFA; values of KMO > 0.60 and significant results in Bartlett’s test were considered acceptable [42]. A parallel analysis [43] was conducted to provide empirical guidance for the number of factors to retain. Parallel analysis creates a random dataset with the same number of cases and variables as the actual dataset with support to retain the factors for which eigenvalues (*λ*) from the actual data are greater than those from the randomly generated data [44]. Extracted components in the EFA were judged to be adequate when their eigenvalues exceeded 1.0 (Kaiser’s criterion) and after visual examination of the scree plot. In addition, items were retained if they had an item-factor loading of at least 0.40 on a primary factor and cross-loadings < 0.25 on other factors [45].

For the second split-half sample, due to the lack of multivariate normality (Mardia’s Skewness 4138.08, *p* < 0.001), CFAs were based on a robust maximum likelihood estimation method with the Satorra-Bentler *χ*^2^ scaled correction [46]. To ensure an identified model, items were set to load freely except for one item per factor, which was set to 1. Model fit was assessed using the following robust indices: comparative fit index (CFI), Tucker–Lewis index (TLI), root mean square error of approximation (RMSEA) and its 90% confidence interval (CI), and standardized root mean square residual (SRMR). Following Gana and Broc, (2019), values of CFI and TLI between 0.90 and 0.95, RMSEA values between 0.06 and 0.08, and SRMR values < 0.08 were indicative of adequate fit [47].

Additionally, modification indices (M.I.) were considered for model improvement and to identify potential misspecifications; M.I. values > 5.0 were assumed to have a significant effect on the model. In addition to M.I., items residuals from the same factor were allowed to correlate also based on theoretical and substantive meaning [38]. A scaled Chi-square difference test (Δ*χ*^2^) was used to compare the original and re-specified models [48]. Given the Likert-type nature of the data, the Omega coefficient and its 95% CI [49] were calculated to determine internal consistency; according to Najera Catalan (2019), values close to 0.80 were considered acceptable [50]. Due to the lack of multivariate normality among measures, the Spearman correlation coefficient was used for evaluating associations across variables. Values of > 0.10–0.29 were considered small, > 0.30–49 were considered moderate, and > 0.50 were considered large correlations [51]. Across the first and second split-half subsamples, all items were subjected to item analysis; no values < 0.20 were expected between latent variables and each of their correspondent items [52].

As sensitivity analyses, *Mann–Whitney U* Rank tests for group comparisons were conducted between participants from the first split-half and second split-half subsamples, in key sociodemographic variables (age and BMI) and across MDDI subscales, to determine if randomization produced equivalent groups. The coefficient *r* (*r* = *z*/square root of N) was used to report the effect size for continuous variables [53]. Cohen's *r* values ≥ 0.10 were considered a “weak” effect, *r* ≥ 0.30 a “moderate” effect, and *r* ≥ 0.50 a “strong” effect. Finally, a two-tailed *p* < 0.05 was considered significant.

R software (version 3.4.4) and the following packages were used: *Psych* [54], *MissMech* [55], *Mice* [56], *MVN* [57], *hornpa* [58], *GPArotation* [59], *MBESS* [60], *Hmisc* [61], *WebPower* [62], *Lavaan* [63], and *semPlot* [64].

## Results

### Exploratory factor analysis

An EFA for the first split-half subsample of gender-expansive participants (*n* = 515) was conducted. The KMO index was 0.75; the Bartlett’s test of sphericity was significant (*χ*^2^(78) = 2856.86, *p* < 0.001); and the mean item communality of 0.53 was > 0.50, suggesting that data and sample size were adequate for the analysis. Results from parallel analysis suggested the presence of three factors; only the first three eigenvalues from the observed data presented *λ* greater than the criterion *λ* (*λ*_1_ = 3.33 > 1.34; *λ*_2_ = 1.93 > 1.90; *λ*_3_ = 3.00 > 1.25); the fourth factor derived from the actual data had a *λ* that was lower than the corresponding criterion *λ* generated by the parallel analysis (*λ*_4_ = 0.82 < 1.42). A three-factor solution for the EFA that accounted for the 53.38% of the variance was observed. Table 1 shows factor loadings, communalities, eigenvalues, and explained variance. Primary factor item-factor loadings ranged from 0.48 to 0.89 across factors (above the 0.40 suggested threshold) with cross-loadings < 0.25 on other factors. Item communalities ranged from 0.27 to 0.78.

**Table 1:** Factor loadings for the exploratory factor analysis in the first split-half subsample of gender-expansive participants (n = 515) from The PRIDE Study

Item/factor	Gender-expansive participants (*n* = 515)			h2
	Factor loadings			
	1	2	3	
Drive for size				
1	**0.79**	− 0.01	− 0.01	0.65
4	**0.89**	0.01	− 0.02	0.78
5	**0.48**	0.21	0.09	0.27
6	**0.60**	0.01	− 0.05	0.35
8	**0.67**	0.03	15	0.49
Appearance intolerance				
2	0.01	**0.62**	0.02	0.39
3	0.07	**0.79**	− 0.04	0.60
7	− 0.20	**0.69**	0.09	0.63
9	0.09	**0.60**	− 0.05	0.34
Functional impairment				
10	0.10	0.15	**0.69**	0.55
11	− 0.08	− 0.12	**0.79**	0.59
12	0.12	0.09	**0.77**	0.66
13	− 0.06	− 0.05	**0.80**	0.62
Eigenvalue	3.33	1.93	3.00	–
Explained variance	.19.88	15.06	18.44	–
Mean item communalities	–	–	–	0.53

### Confirmatory factor analysis

**Fig. 1:**
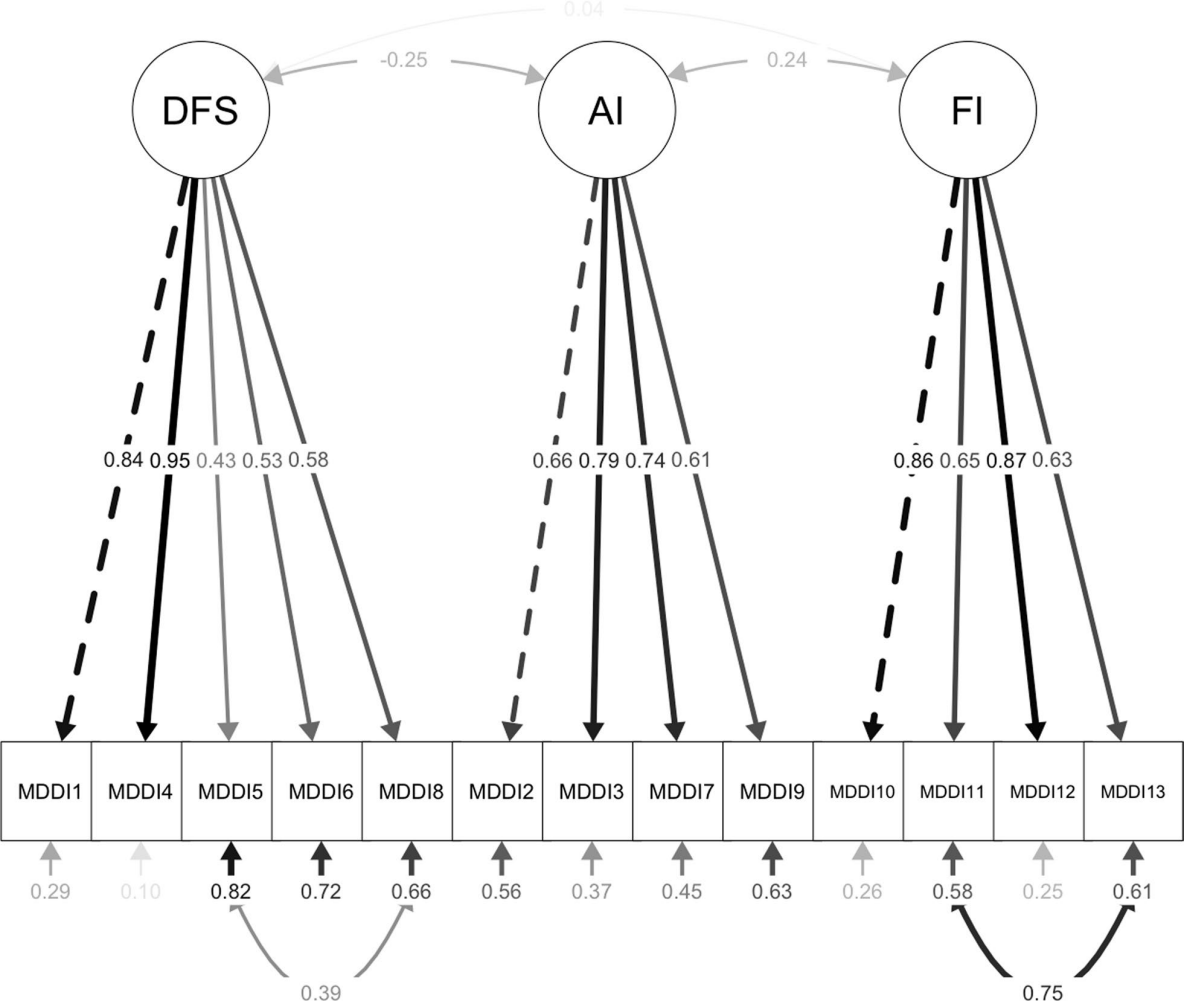
Confirmatory factor analysis of the re-specified retained 3-factor model for the Muscle Dysmorphic Disorder Inventory (MDDI) in the second split-half subsample of gender-expansive participants (N = 516). *Note: DFS* Drive for Size factor, *AI* Appearance Intolerance factor, *FI* Functional Impairment factor

A CFA using the EFA model derived from the first split-half subsample was then conducted using data from the second split-half subsample of gender-expansive participants (*n* = 516). Fit indices were marginally below the suggested threshold (see Table 2); however, an inspection of the M.I. revealed high correlations between items 11 (“I pass up social activities with friends because of my workout schedule”) and 13 (“I pass up chances to meet new people because of my workout schedule”) (M.I.: 209.73) from the MDDI FI subscale, and between items 5 (“I think my chest (muscle) is too small”) and 8 (“I wish my arms were bigger”) (M.I.: 75.86) from the MDDI DFS subscale. Therefore, the model was re-specified allowing for residuals to correlate (see Table 2). The respecified model showed adequate fit and significantly improved the model fit (Δ*χ*^2^(2, *n* = 516) = 160.47, *p* < 0.001). Figure 1 shows standardized parameters (factor loadings and factor correlations) for the respecified model. All factor loadings were statistically significant (*ps* < 0.001) and > 0.30 (standardized parameters).

**Table 2:** Robust fit indices values for the tested models in the second split-half sample of gender-expansive participants (n = 516) from The PRIDE Study

Models	CFI	TLI	RMSEA [CI 90%]	SRMR
1. MDDI	0.81	0.76	0.13 (0.12, 0.14)	0.09
2. MDDI Re-specified	0.94	0.92	0.08 (0.07, 0.09)	0.07

### Internal consistency, convergent validity, and item analyses

Table 3 reports descriptive statistics, Omega values with 95% CI, and Spearman correlations among variables within both the first and second split-half subsamples. The Omega coefficient for the MDDI subscales ranged between 0.77 and 0.86 across the two samples, providing support for the internal consistency of the subscales. With regard to convergent validity, the MDDI AI subscale had significant, large positive correlations with the EDE-Q Shape Concern and Weight Concern subscales in both split-half subsamples as expected (*r*_*s*_ = 0.63–0.79, *p*s < 0.01). The MDDI AI subscale was further found to have significant, positive correlations (moderate to large) with the other EDE-Q subscales and the Global Score in both subsamples (*r*_*s*_ = 0.41–0.78, *p*s < 0.01). Also consistent with expectations, the MDDI FI subscale had significant, moderate positive correlations with the EDE-Q Global Score in both split-half subsamples (*r*_*s*_ = 0.33–0.36, *p*s < 0.01). The MDDI FI subscale additionally showed significant, positive correlations (small to moderate) with all of the EDE-Q subscales in both subsamples (*r*_*s*_ = 0.27–0.37, *p*s < 0.01). Finally, as anticipated, the MDDI DFS subscale showed significant, small negative correlations with the EDE-Q Weight Concern subscale in both split-half subsamples (*r*_*s*_ = − 0.20–0.24, *p*s < 0.01) and with the EDE-Q Restraint subscale in the second split-half subsample (*r*_*s*_ = − 0.09, *p* < 0.05); the correlation with EDE-Q Restraint in the second split-half subsample was similar in size but non-significant (*r*_*s*_ = − 0.07, *p* > 0.05). The MDDI DFS subscale additionally showed significant, small negative correlations with all of the EDE-Q subscales and the Global Score in both subsamples (*r*_*s*_ = − 0.08 to − 0.24, *p*s < 0.05).

**Table 3:** Internal consistency, descriptive statistics, and Spearman correlations in the first and second split-half subsamples of gender expansive participants (N = 1031) from The PRIDE Study

		First split-half sample of gender expansive participants		Second split-half sample of gender expansive participants		1	2	3	4	5	6	7	8
		(n = 515)		(n = 516)									
		Omega (95% CI)	*M* (*SD)*	Omega (95% CI)	*M* (*SD)*								
1	MDDI DFS	0.80 (0.76, 0.83)	8.27 (3.83)	0.81 (0.76, 0.86)	8.31 (3.87)		− 0.18**	0.13**	− 0.09*	− 0.08*	− 0.24**	− 0.16**	− 0.18**
2	MDDI AI	0.77 (0.73, 0.80)	12.92 (3.95)	0.80 (0.77, 0.82)	12.71 (4.13)	− 0.11*		0.17**	0.48**	0.63**	0.75**	0.80**	0.78**
3	MDDI FI	0.86 (0.78, 0.90)	6.10 (3.08)	0.81 (0.76, 0.86)	6.20 (3.10)	0.21**	0.18**		0.37**	0.28**	0.30**	0.29**	0.33**
4	EDE-Q R	0.85 (0.83, 0.86)	1.28 (1.49)	0.86 (0.83, 0.88)	1.24 (1.49))	− 0.07	0.41**	0.36**		0.57**	0.65**	0.64**	0.77**
5	EDE-Q EC	0.85 (0.82, 0.87)	1.03 (1.26)	0.86 (0.82, 0.88)	1.02 (1.28)	− 0.09*	0.55**	0.34**	0.55**		0.73**	0.75**	0.81**
6	EDE-Q WC	0.85 (0.83, 0.87)	2.24 (1.57)	0.87 (0.85, 0.88)	2.15 (1.69)	− 0.20**	0.73**	0.27**	0.58**	0.69**		0.91**	0.95**
7	EDE-Q SC	0.90 (0.88, 0.91)	2.64 (1.61)	0.91 (0.90, 0.92)	2.55 (1.69)	− 0.08*	0.79**	0.32**	0.56**	0.67**	0.86**		0.96**
8	EDE-Q G	0.94 (0.93, 0.95)	1.95 (1.32)	0.95 (0.94, 0.96)	1.89 (1.41)	− 0.12**	0.76**	0.36**	0.74**	0.78**	0.92**	0.94**	

*MDDI DFS* MDDI Drive for Size subscale, *MDDI AI* MDDI Appearance Intolerance subscale, *MDDI FI* MDDI Functional Impairment subscale, *EDE-Q R* EDE-Q Restraint subscale, *EDE-Q EC* EDE-Q Eating Concern subscale, *EDE-Q WC* EDE-Q Weight Concern subscale, *EDE-Q SC* EDE-Q Shape Concern subscale, *EDE-Q G* EDE-Q Global Score

Correlations for the first split-half sample are located below the diagonal. Correlations for the second split-half sample are located above the diagonal

*p < 0.05

**p < 0.01

In both split-half subsamples, item analyses revealed strong significant positive correlations between items and their latent factor for the MDDI DFS (*r*_*s*_ = 0.51 to 0.82, *ps* < 0.001), MDDI AI (*r*_*s*_ = 0.73 to 0.83, *ps* < 0.001), and MDDI FI (*r*_*s*_ = 0.60 to 0.94, *ps* < 0.001) subscales.

### Sensitivity analyses

The *Mann–Whitney U* test was used to assess differences in key sociodemographic variables (age and BMI) and across all subscales of the MDDI. No significant differences were observed between the first and second split-half samples of gender-expansive participants on age (*Mann Whitney U* test: *z* = 0.74, *p* = 0.770, Cohen’s *r* = 0.02), BMI (*Mann Whitney U* test: *z* = − 0.88, *p* = 0.190, Cohen’s *r* = 0.03), or across MDDI subscales (DFS: *Mann Whitney U* test: *z* = 1.64, *p* = 0.950, Cohen’s *r* = 0.05; AI: *Mann Whitney U* test: *z* = − 0.12*, p* = 0.451, Cohen’s *r* = 0.01; FI: *Mann Whitney U* test: *z* = − 0.2, *p* = 0.493, Cohen’s *r* = 0.01), suggesting that the randomization process was effective in balancing the groups.

## Discussion

This study is one of only a few to focus on muscle dysmorphia symptoms in a sample of gender-expansive individuals, and further represents the first psychometric evaluation of the MDDI in this potentially at-risk, yet understudied population. Despite elevated body dissatisfaction [16] and eating disorder symptomatology [15] among gender minorities, gender-expansive people have been largely underrepresented in muscle dysmorphia research. Our goal was to validate the MDDI in a sample of gender-expansive people to encourage future research on muscle dysmorphia in this population. Broadly, our analyses supported the original three-factor structure of the MDDI, and the subscales were found to show adequate internal consistency and convergent validity based on associations with a theoretically relevant measure of disordered eating.

Using an exploratory and confirmatory factor analytic approach, we found support for the original three-factor structure of the MDDI, suggesting the distinct nature of the three subscales that assess different dimensions of muscle dysmorphia symptomatology. This finding is consistent with previous MDDI validation studies in various samples from a broad array of countries (e.g., Argentina, Turkey, Germany, Brazil) that have confirmed the original three factors proposed by Hildebrandt et al. [22–28]. Although these studies have predominantly been conducted in men (cisgender or presumed cisgender) with very little attention to sexual or gender minority status, a recent study replicated the three-factor structure in cisgender gay men and lesbian women [29].

In terms of MDDI subscale intercorrelations, consistent with previous findings, we found that the Functional Impairment subscale was positively correlated with the Appearance Intolerance and Drive for Size subscales in both split-half subsamples [20, 22, 27, 29]. There were, however, significant negative correlations between the Drive for Size and Appearance Intolerance subscales in both subsamples. This latter finding diverges from previous validation studies conducted in cisgender or presumed cisgender men [20, 22, 28]. However, there were negative associations between these two subscales in one study of a sample of approximately half women (though gender identity was not specifically assessed) [26] and another conducted in cisgender sexual minority men and women [29]. This may suggest that, in cisgender women and sexual and gender minority people, the drive to be thin and lean is more central to concerns about appearance than increased body size. However, additional research is needed to confirm these findings and better understand the underlying mechanisms.

Consistent with our hypotheses, the MDDI subscales showed adequate internal consistency reliability in this sample of gender-expansive individuals. As part of the convergent validity evaluation, we found expected moderate to large positive correlations of the MDDI Appearance Intolerance and Functional Impairment subscales with all of the EDE-Q subscales and the global score in both split-half subsamples. In line with our hypotheses, the MDDI Drive for Size subscale was negatively correlated with EDE-Q subscale and global scores in both split-half subsamples with one exception (i.e., similar effect size, but non-significant correlation with the EDE-Q restraint in the first subsample). These findings are generally consistent with the patterns of associations between the MDDI and measures related to disordered eating that have been reported in previous studies including in cisgender gay men and lesbian women [29]. However, other studies, including one in Brazilian men and one in Argentinian men (though gender identity was not specifically assessed), found that MDDI Drive for Size and EDE-Q scores were positively associated [22, 28]. More research is needed to understand the nature of associations between muscle dysmorphia symptoms and disordered eating across diverse populations.

### Strengths and limitations

Strengths of this study include a large sample size from a national cohort, the focus on an understudied population of gender minority people, and distinguishing gender-expansive from binary transgender people (i.e., transgender men [65] and transgender women), as these two groups have been found to differ in their body image concerns [15, 19]. However, future research should evaluate specific gender-expansive subpopulations (e.g., non-binary, agender, gender fluid) as our gender-expansive group was diverse and included combinations and write-ins, such that there were insufficient sample sizes for conducting specific gender-expansive subgroup analyses. Additional limitations should be addressed. First, the sample was comprised of English-speaking, predominantly White, and highly educated individuals, therefore limiting the generalizability of the findings. Given that nearly one-third of gender-expansive people are not White [66], gender-expansive people of color are underrepresented in the present study, and their experiences with body image and muscle dysmorphia symptoms may differ from those of most of our study participants. Future studies of these constructs will be needed in samples reflecting greater racial, ethnic, and socioeconomic diversity. Second, the MDDI is a measure of the severity of muscle dysmorphia symptoms, but it cannot provide a diagnosis for the disorder, which would require a full, structured interview. Third, data were not available to evaluate the test–retest reliability or the discriminant validity of the MDDI subscales since all constructs measured were likely to be correlated in this investigation; these important psychometric properties should be investigated in future research. Finally, the conceptually relevant overlap between the constructs of muscle dysmorphia symptoms and disordered eating supported using the EDE-Q for evaluating convergent validity evaluation in this study; however, future studies should further examine convergent validity based on associations of the MDDI subscales with measures of other theoretically related constructs, including muscularity-oriented disordered eating (e.g., the Muscularity Oriented Eating Test) [67], body dysmorphic disorder symptoms (e.g., the Appearance Anxiety Inventory [68], the Body Dysmorphic Disorder Questionnaire) [69], and other key diagnostic features (e.g., body checking and avoidance).

## Conclusions and future directions

In sum, this study supports the three-factor structure of the MDDI and the psychometric properties of the subscales in gender-expansive individuals. The support found for the MDDI in this study will facilitate its use in future research on muscle dysmorphia symptoms among gender-expansive individuals, who are at elevated risk for a variety of eating- and body image-related concerns. Future studies will be needed to further evaluate other psychometric properties of the MDDI in this population, including test-rest reliability and discriminant validity, as well as investigating predictive validity using longitudinal data. Further, building upon work that has established MDDI norms in gender-expansive people [19], additional studies will be needed to develop clinical cut-off scores for the MDDI and to establish comprehensive prevalence estimates for muscle dysmorphia in gender minority groups. Finally, it will be important to explore the specific nature and diversity of body image ideals in gender-expansive people, especially those related to the drive for size and muscularity, and to consider how these may influence the risk for muscle dysmorphia.

## Data Availability

Data from The PRIDE Study may be accessed through an Ancillary Study application (details at pridestudy.org/collaborate).

## Abbreviations

AI: Appearance intolerance
CFA: Confirmatory factor analysis
CI: Confidence interval
DFS: Drive for size
EDE-Q: Eating Disorder Examination-Questionnaire
EFA: Exploratory factor analysis
FI: Functional impairment
MDDI: Muscle dysmorphic disorder inventory
MI: Modification indices
PRIDE Study: Population research in identity and disparities for equality
RMSEA: Root mean square error of approximation
SRMR: Standardized root mean square residual
TLI: Tucker–Lewis index
